# Surveillance of Smoldering Myeloma Patients Who Progress to Active Disease Is Associated with Favorable Outcomes

**DOI:** 10.3390/cancers18040658

**Published:** 2026-02-18

**Authors:** Gil Fridberg, Inbar Cohen, Renana Robinson, Iuliana Vaxman, Tamir Shragai, Svetlana Trestman, Tomer Ziv-Baran, Natan Melamed, Pia Raanani, Irit Avivi, Yael C. Cohen

**Affiliations:** 1 Hematology Division, Tel Aviv Sourasky Medical Center, Tel Aviv 6423906, Israel; gilfr@tlvmc.gov.il (G.F.); tamirsh@tlvmc.gov.il (T.S.); svetlanat@tlvmc.gov.il (S.T.); natanm@tlvmc.gov.il (N.M.); iritavi@tlvmc.gov.il (I.A.); 2 Gray Faculty of Medical & Health Sciences, Tel Aviv University, Tel Aviv 6997801, Israel; inbarco1@clalit.org.il (I.C.); renana.robinson@clalit.org.il (R.R.); juliava1@clalit.org.il (I.V.); praanani@012.net.il (P.R.); 3 Hematology Institute, Davidoff Cancer Center, Rabin Medical Center, Petah Tikva 6356710, Israel; 4 School of Public Health, Faculty of Medical & Health Sciences, Tel Aviv University, Tel Aviv 6997801, Israel; zivtome@tauex.tau.ac.il

**Keywords:** multiple myeloma, smoldering multiple myeloma

## Abstract

Introduction: Smoldering multiple myeloma (SMM) is a precursor condition to active multiple myeloma (MM). While surveillance is standard, data on its effectiveness in preventing irreversible complications is limited. Methods: We conducted a two-center matched retrospective cohort study comparing 57 patients who progressed from SMM (pSMM) with 57 de novo MM (dnMM) patients, matched by age and diagnosis year. Primary endpoints were end-organ damage and progression-free survival (PFS). Results: Median surveillance prior to progression in pSMM was 40 months. At active MM diagnosis, irreversible renal/bone complications were observed in 44% of pSMM patients compared with 72% of dnMM (*p* = 0.002). dnMM patients presented more frequently with hypercalcemia (12.3% vs. 1.8%, *p* = 0.03), lytic lesions (67% vs. 45%, *p* = 0.02), pathological fractures (31% vs. 10%, *p* = 0.008), and detrimental bone disease (58% vs. 25%, *p* < 0.001). ISS and R-ISS stages were higher in dnMM, while treatment patterns were similar. At 3 years, PFS was significantly higher in pSMM (59% vs. 30%, *p* = 0.01), as was overall survival (OS; 92% vs. 76%, *p* = 0.01). Clinical surveillance was associated with reduced disease burden, fewer irreversible complications, and improved PFS and OS compared with dnMM. Conclusion: These findings support surveillance effectiveness and highlight the need to optimize follow-up strategies.

## Simple Summary

Smoldering multiple myeloma (SMM) is an asymptomatic precursor to active multiple myeloma (MM). Standard management involves surveillance to detect progression before severe organ damage occurs. We compared clinical outcomes between 57 patients who progressed from monitored SMM and 57 matched patients diagnosed with de novo MM. Patients previously under surveillance presented with lower disease burden and fewer irreversible complications, such as renal failure and bone fractures (44% vs. 72%), compared to the de novo group. Furthermore, the surveillance cohort showed superior 3-year progression-free survival (59% vs. 30%) and overall survival (92% vs. 76%). These findings suggest that diagnosis via clinical surveillance is linked to reduced disease severity and favorable survival outcomes. However, since organ damage still occurred in monitored patients, follow-up strategies require optimization to better prevent complications.

## 1. Introduction

Smoldering Multiple Myeloma (SMM) is an intermediate precursor phase in the evolution of multiple myeloma (MM). According to consensus guidelines, patients with SMM are monitored for progression to active disease, assuming such surveillance will identify myeloma-defining events (MDEs) early and consequently hasten initiation of treatment, prevent complications and improve outcomes [1,2].

Several clinical, biochemical and genetic characteristics were identified as risk factors for progression and aid in stratification of patients and guide management [3,4]. Although previous and more recent studies demonstrated the benefit of treating high-risk SMM patients with lenalidomide [5,6] or with daratumumab [7], for the vast majority of patients, surveillance remains the mainstay approach [1,8,9].

However, it is not yet established to what extent this approach is successful in preventing detrimental organ damage. While favorable survival has been reported for patients with preceding SMM [10] or MGUS (monoclonal gammopathy of undetermined significance) [11] compared to patients with de novo myeloma, data describing the presentation, clinical course and outcome in SMM patients progressing to active MM is scarce and not comparable to dnMM [4,12,13].

We aimed to investigate the effectiveness of real-life SMM clinical surveillance by comparing patients who presented with active myeloma who underwent clinical surveillance for a preceding diagnosis of SMM, to patients who presented with active myeloma without a known prior precursor condition of SMM or of MGUS. We focused on the extent of end-organ involvement as well as outcomes of first-line therapy. Additionally, we investigated the association between surveillance patterns and outcomes in SMM patients progressing to active MM.

## 2. Methods

We performed a two-center retrospective, matched-cohort study. All consecutive SMM patients who progressed to active MM (pSMM) and initiated anti-myeloma therapy between September 2008 and February 2023 were compared with a cohort of myeloma patients who presented with active disease during that period without a known preceding diagnosis of precursor condition (SMM or MGUS), termed de novo patients (dnMM). To form the de novo cohort, we utilized a sequential matching strategy in which each patient in the pSMM group was matched with the first available patient from the de novo set, based on two specific criteria: age and year of diagnosis (±2 years).

Eligibility criteria included age ≥ 18 years, newly diagnosed active MM requiring and leading to treatment initiation according to IMWG criteria (CRAB or SLiM-CRAB) for the SMM cohort, the surveillance period was defined as the timeframe commencing after the completion of the baseline work-up (comprising bone marrow assessment, imaging, and at least two longitudinal laboratory evaluations) and ending at the diagnosis of active multiple myeloma. A minimum of three months of surveillance following this confirmed diagnosis was required for inclusion in the pSMM group. Patients who received treatment for SMM and those diagnosed before 2016 who retrospectively fulfilled SLiM criteria were excluded from the cohort. Patients in the dnMM cohort were defined as those with no documented history of MGUS or SMM, and who were not enrolled in any hematological surveillance program prior to their active disease diagnosis. Clinical records between September 2008 and February 2023 were screened, and the data was extracted in July 2023. All medical records were manually reviewed to confirm eligibility and extract characteristics, treatments, and outcomes. The study protocol was reviewed and approved by the Institutional Review Board.

SMM, MM, MDEs, staging and treatment response were defined according to the International Myeloma Working Group (IMWG) criteria [1,14]; creatinine clearance was calculated using the CKD-EPI equation from 2021 [15].

Progression-free survival (PFS) and overall survival (OS) periods were calculated from the date of diagnosis of active MM to the earliest of the following: occurrence of the event or end of follow-up. Detrimental bone disease was defined as a pathological fracture and/or ≥3 lytic lesions; this arbitrary threshold was used to categorize high-burden skeletal involvement. Potentially irreversible MDEs were defined as renal injury (Cr > 2 mg/dL or creatinine clearance ≤ 40 mL/min) and/or detrimental bone involvement.

The categorical parameters were summarized as numbers and percentages. The continuous variables distribution was evaluated using a histogram, and they were reported as median and interquartile range. Categorical variables were compared between groups using the Chi-square test or Fisher’s exact test, and continuous variables were compared using the Mann–Whitney test. Reverse censoring was used to evaluate the length of follow-up. Kaplan–Meier curves and Log-rank tests were used to study the association between the group and PFS and OS. All statistical tests were two-sided, and *p* < 0.05 was considered significant. Data was analyzed using the IBM SPSS version 29.0.2.

This study was performed in accordance with the Declaration of Helsinki, and the study was approved by the local Institutional Review Board (IRB).

## 3. Results

### 3.1. Patients

Fifty-seven consecutive patients who were diagnosed with SMM and progressed to active MM fulfilled the inclusion criteria to form the post SMM cohort. The pSMM was compared with 57 matched de novo MM patients (dnMM cohort). Baseline demographic and clinical characteristics of patients are presented in Table 1.

Median SMM surveillance period (from SMM diagnosis to active MM) was 40 months (range 3–167). Clinic visits were 4.02 (IQR 2.72–5.54) visits/year, and imaging studies frequency was 1.73 (IQR 0.83–3.53) studies/year, both of which are consistent with IMWG consensus recommendations.

Median follow-up from time of active MM diagnosis was 39 months (range 0.9–169) in the pSMM cohort, and 43 months (range 0.3–180) in the dnMM cohort. There were no differences between the cohorts in baseline demographics of gender (*p* = 0.09) and median age (*p* = 0.94). Median time to progression from SMM diagnosis to active MM was 25 months (range 3–167).

### 3.2. Disease Characteristics and Myeloma-Defining Events

ISS and R-ISS were defined at active disease diagnosis. The rate of high-risk cytogenetics at diagnosis was similar between groups (*p* = 0.82); dnMM patients had a higher ISS/R-ISS score at presentation compared with pSMM patients: ISS ≥ II (67% vs. 33%, respectively, *p* = 0.003) and R-ISS ≥ II (84% vs. 46%, respectively, *p* = 0.001). Additional disease characteristics were balanced between the cohorts: heavy and light chain type (*p* = 0.11, *p* = 0.68, respectively), M-protein levels (*p* = 0.28), oligo-secretory disease (5.2% vs. 8.7%, *p* = 0.72) and the incidence of extramedullary disease (*p* = 0.19).

More patients in the dnMM group presented with hypercalcemia, 12.3% vs. 1.8% (*p* = 0.03), and similarly, the presence of any lytic lesion at diagnosis was more frequent in the dnMM group vs. the pSMM cohort, 67% vs. 45% (*p* = 0.02), respectively. There was a trend towards a higher rate of bone marrow plasma cell infiltration (BMPC) ≥ 60% in dnMM (*p* = 0.057). Other myeloma-defining events were similar between groups: anemia (*p* = 0.8), renal failure (*p* = 0.4) and FLCr ≥ 100 (*p* = 0.67).

Higher levels of involved light chain were associated with renal disease at presentation in the dnMM cohort (median 698 mg/L with renal impairment vs. 158 mg/L in patients with no renal impairment, *p* = 0.03), but not in the pSMM cohort (*p* = 0.31).

### 3.3. Myeloma-Defining Events

De novo MM patients were more likely to experience CRAB as an MDE compared to pSMM patients (93% vs. 79%, *p* = 0.04). De novo MM patients experienced a significantly higher rate of pathological fractures compared with pSMM patients, 31% vs. 10% (*p* = 0.008), and more dnMM patients suffered from bone pain, 55% vs. 26% (*p* = 0.002), as assessed qualitatively during clinical visits. Detrimental bone involvement occurred more frequently in dnMM patients compared with pSMM patients, 58% vs. 25% (*p* < 0.001).

**Figure 1:**
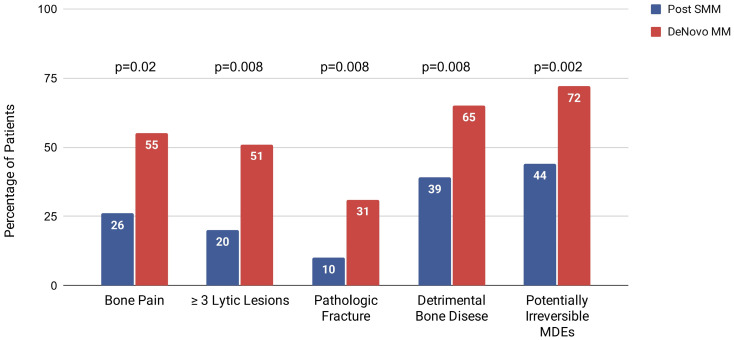
Bone disease and potentially irreversible MDEs at diagnosis of active MM.

Potentially irreversible MDEs reflected by the composite outcome of renal involvement and/or detrimental bone disease were also more frequent in the dnMM vs. pSMM cohort, 72% vs. 44% (*p* = 0.002) (Figure 1).

In the pSMM cohort, 11/57 patients had at least one lytic lesion, 14/57 patients had a detrimental bone disease, and 9/57 patients had renal involvement. Altogether, only 25/57 (44%) patients had no renal and/or bone involvement as an MDE.

### 3.4. Treatment and Response

Treatment patterns and response are summarized in Table 2. Initiation of anti-myeloma treatment during an acute inpatient hospitalization occurred more frequently in the dnMM group compared with pSMM, 33% vs. 12% (*p* = 0.01), respectively. Treatment patterns were mostly balanced between groups, except for the use of bortezomib, which was more frequent in the dnMM cohort (*p* = 0.007).

**Figure 2:**
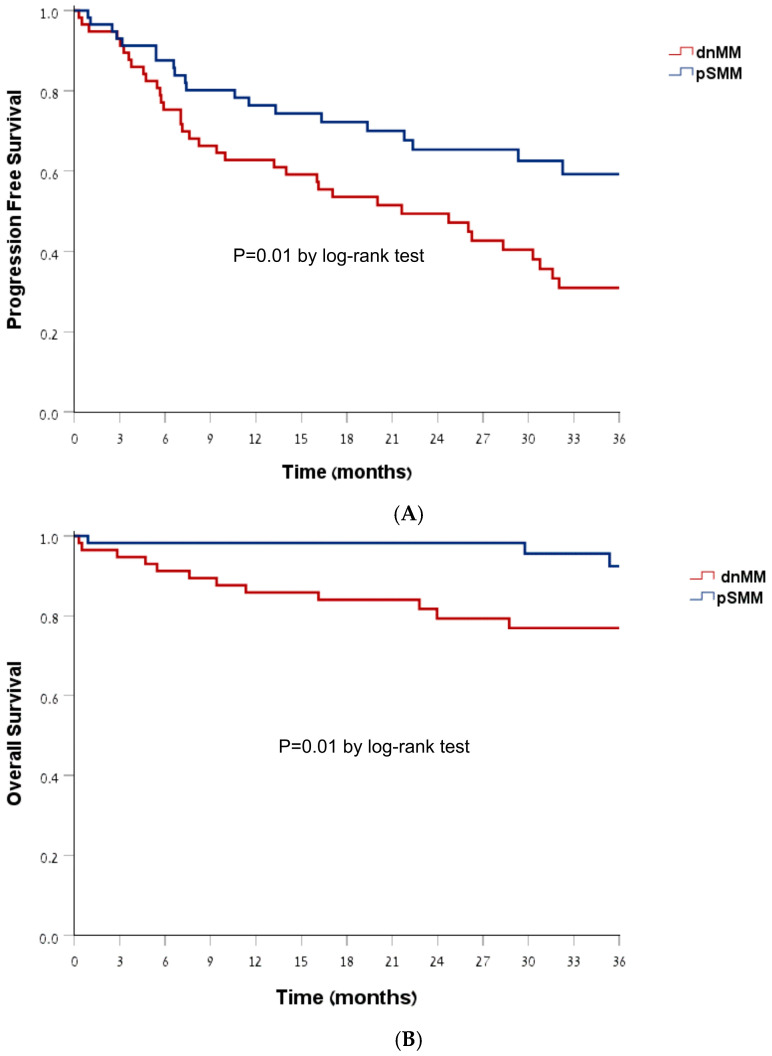
Kaplan–Meier estimates of survival. Panel (**A**) Progression-free survival, Panel (**B**) Overall survival.

Overall response rate (ORR) to first-line treatment was comparable between groups (*p* = 1.0), as was achieving a response equal to or higher than VGPR (Very Good Partial Response), 56% in both groups (*p* = 0.88). At 3-year follow-up, pSMM patients had superior PFS compared with dnMM: 59% vs. 30%, respectively (Log Rank 0.01) (Figure 2). OS was also higher in the pSMM group at 3-year follow-up, 92% vs. 76% in the dnMM (Log Rank 0.01). Differences in PFS and OS were similar when adjusted for year of diagnosis, age, gender, ISS and R-ISS.

## 4. Discussion

Clinical surveillance remained, until recently, the recommended standard of care for patients diagnosed with SMM. The underlying premise is that careful monitoring of patients’ symptoms, laboratory values and particular disease biomarkers will allow early detection of those patients who develop active disease, at a time when target organ damage is limited or absent. Yet these recommendations are based on expert consensus, and evidence is lacking as to the extent to which this practice improves patients’ outcomes. Such data may provide insight for affirmation of current practice, or alternatively, a need for intensification of SMM patient surveillance or consider earlier intervention. This is particularly important in light of recent data from the AQUILA study [7], which demonstrated a progression-free survival (PFS) benefit in treating high-risk smoldering multiple myeloma (SMM) patients with single-agent anti-CD38 antibody therapy. Understanding the natural history of SMM and the impact of surveillance in an era where early treatment confers benefit may help refine patient selection strategies aimed at improving outcomes and preventing end-organ damage.

In this retrospective matched-cohort study, we demonstrated that patients who developed active myeloma during SMM surveillance (pSMM) experienced a less aggressive clinical phenotype than a matched cohort of patients who presented with active myeloma, without having been identified and monitored for a preceding precursor condition (dnMM). Additionally, with a median follow-up of 39 months, pSMM patients experienced favorable PFS and OS compared to their dnMM counterparts.

The cohorts were comparable in terms of median age, which was characteristic of NDMM patients [16]. The rate of high-risk cytogenetics was similar between pSMM and dnMM cohorts. ISS score distribution was similar in the dnMM group to that previously described [17], though the R-ISS score was higher compared with previous studies [18,19]. In the pSMM group, both ISS and R-ISS were lower compared with dnMM, despite comparable cytogenetic risk. This is probably due to the lower disease burden upon conversion to active disease in the pSMM cohort, reflecting effective surveillance.

Bone disease occurs in up to 60–80% of MM patients at diagnosis [8,20], with potentially debilitating implications [21]. We observed similar rates to those previously reported in the de novo cohort. Despite clinical surveillance, a non-negligible proportion of pSMM patients still developed bone disease, yet this occurred at a significantly lower rate than the dnMM patients. In both clinical practice and research, bone disease is traditionally defined in a dichotomic manner for diagnosis [1], although a greater extent of bone disease was shown to be associated with worse prognosis [22]. We observed favorable rates in pSMM patients in specified bone involvement patterns, which, based on our clinical experience (consensus between authors: IV, YCC, IA, TS), we assessed as having clinical impact, namely: bone pain, lytic lesions, pathologic fracture, and composition of all. Reduced bone disease in the pSMM cohort may reflect effective monitoring, allowing for diagnosis of active myeloma at the biochemical progression stage, prior to target organ damage. From a clinical perspective, it is anticipated that awareness towards lytic bone disease is higher among patients with SMM, compared with the general population, which is likely to lead to earlier detection of lytic bone disease, while it is still limited in its severity and extent. These findings are also in line with the pathogenesis of bone disease [20] that was suggested to be quantitative in nature and correlates with the extent of plasma cell infiltration [23,24], which was indeed significantly lower in the SMM cohort patients. As hypercalcemia is mostly associated with bone disease and vice versa [25], lower rates of hypercalcemia as a presenting feature in the pSMM cohort reflect a lower extent of their bone involvement.

Patients with pSMM had a lower rate of inpatient admissions for treatment initiation compared to those with dnMM, reflecting a more indolent clinical onset. In contrast, hospitalization among dnMM patients was more often associated with an aggressive clinical presentation requiring urgent treatment and inpatient supportive care. This was further supported by higher rates of inpatient surgical-orthopedic interventions, pain management, and antibiotic use for significant infections in the dnMM group.

Renal involvement was numerically lower in the pSMM cohort as in previously reported series [26,27,28,29], although not statistically significant when compared to the dnMM cohort. In the dnMM patients, renal disease was associated with higher involved light chain levels, in contrast to pSMM patients, where such an association was not seen. This may suggest a more typical cast nephropathy pathogenesis of renal injury, stemming from involved light chain load in the dnMM cohort vs. a more multifactorial renal injury in SMM patients during follow-up, with possible various aggravating factors such as dehydration, nephrotoxic drugs and preexisting conditions which affect renal function [30,31,32,33,34,35]. Nevertheless, the similar rates of renal injury at presentation emphasize an unsatisfactory outcome of clinical follow-up and surveillance aimed to prevent such complications.

pSMM patients were significantly less likely to experience CRAB as their MDE (Table 1), yet most (66%) pSMM patients still experienced lytic lesions and/or renal failure as their MDE. While rates are lower compared to dnMM presentation as active disease, nevertheless, they show that clinical follow-up still fails to prevent potentially irreversible and debilitating complications in most patients.

Our cohort spans across a long period of time (2008–2023) in which the landscape of first-line treatment has changed, and is affected by local reimbursement, in particular, with the introduction of the anti-CD38 monoclonal antibody. The cohorts also include both transplant-eligible and ineligible patients. To minimize bias, we matched patients by age and year of diagnosis, so that treatment patterns would be comparable. Indeed, baseline characteristics of patients are mostly similar. Overall response rates and a response greater than or equal to VGPR were comparable between cohorts; however, pSMM patients had statistically significant, favorable PFS and OS at 3 years follow-up, the latter compatible with previous studies [10]. The relatively short PFS observed, compared to that expected in newly diagnosed patients in recent years, may be attributed to the extended study duration and changes in the treatment landscape.

The longer PFS and OS in the SMM cohort might be explained by several factors. Both previous and recent studies have established the concept of biological heterogenicity of plasma cell dyscrasias and its predictive value of aggressive disease phenotype, response to treatment and survival [36,37,38]. Patients with more indolent MM biology may have extended periods of their precursor condition, hence increasing their likelihood of being diagnosed by incidental laboratory tests during their SMM phase, creating a lead time bias. Another explanation for these favorable long-term outcomes may be related to the lower disease burden at the time of initiation of treatment, which may contribute towards lessened therapy-resistance and improved treatment outcomes. Evidence regarding disease burden (i.e., percentage of plasma cells, markers such as beta-2-microglobulin, albumin, LDH) is also considered predictive of prognosis [17,39].

Indeed, in our study, dnMM patients had a higher disease burden at presentation as reflected by greater plasma cell infiltration in bone marrow, and a higher rate of unfavorable ISS and RISS. It is noteworthy that there was no significant difference in other known risk factors between the two cohorts, i.e., cytogenetics, age, and treatment patterns.

Previous retrospective studies have shown an overall survival advantage in patients with precursor conditions who progressed to active MM compared with those with newly diagnosed MM. In a single-institution retrospective matched-control study, reporting data from an earlier period (1973–2015) than our study, 774 patients with prior MGUS, SMM or a solitary plasmacytoma who developed active myeloma were compared to matched controls without a known preceding precursor diagnosis [10]. This study focused on overall survival, which was favorable for the cases vs. controls (71 vs. 56 months). Further analysis showed that benefit was seen in SMM and solitary plasmacytoma patients, but not in the MGUS patients. Cases had lower BMPC%, LDH and beta-2-microglobulin, and a higher rate of ISS3, similar to our findings. In contrast to our findings, this study reported a higher rate of high-risk FISH among controls, and a lower rate of VGPR response; descriptive data on end-organ damage were not included.

Another study, based on a Medicare-linked database, analyzed outcomes of 17,457 patients diagnosed with MGUS between 1994 and 2007 [11]. Analogous to our findings among SMM patients, they found that patients who had MGUS follow-up (6%) compared to those without MGUS follow-up, had significantly fewer complications at MM diagnosis (kidney injury, cord compression, dialysis, fracture, and hypercalcemia) and improved disease-specific survival.

Data on clinical phenotype at active myeloma presentation among 140 SMM patients was recently published, showing that most SMM patients progressing to active MM present SLiM criteria, 43% present with bone lesions and 2% with renal failure; this report lacked comparison to dnMM patients [12,13].

In the AQUILA study, 50% of the patients in the active monitoring arm progressed to active MM. Of these, only 17% had CRAB as their MDE, vs. 33% who had SLiM. Our findings show a much higher rate of CRAB MDE in the pSMM cohort, despite standard clinical surveillance, monitoring patients every 3–4 months. This may reflect differences in real-world vs. clinical trial patient populations, as well as more strict monitoring within a clinical trial setting, which may be challenging to achieve with real-world resources. These observations support the need to further explore whether refining surveillance strategies—such as increasing visit frequency or incorporating routine imaging could enhance outcomes and potentially offer a less intensive alternative to early treatment initiating in selected patients. On the other hand, while some criticize the benefit of treatment of high-risk SMM with daratumumab based on AQUILA findings, arguing that the rate organ damage observed in the active monitoring group was relatively low, our findings suggest that in the real world, rates of CRAB among SMM patients who develop active disease are much higher, possibly they could be reduced or prevented with daratumumab treatment.

We acknowledge several limitations of this study, mainly a relatively small sample size and a retrospective design. We also acknowledge that our analysis lacked data on non-myeloma comorbidities and socioeconomic factors. Although age-matching provides some control for general health trends, the lack of specific data on these potential confounders represents a limitation in our ability to fully assess their impact on overall survival. Noteworthy that all patients in this study were managed at two major medical centers located in the same geographic region. The regional proximity of the two participating sites, combined with mandatory universal coverage under the Israeli National Health Insurance Law, ensures that both cohorts had access to the same specialized hematological services and referral networks. These factors may help mitigate the risk of socioeconomic or geographic segregation between the groups. Nevertheless, we acknowledge that unmeasured cultural factors could still influence medical engagement. The difference in treatment patterns is also notable with respect to outcomes. Regarding bortezomib administration, higher use in the dnMM group might be attributed to higher rates of renal involvement (although not statistically significant). Also worth mentioning is the higher numerical use of daratumumab in the pSMM group, though this difference was mild and not statistically significant.

## 5. Conclusions

In conclusion, our data suggest that despite clinical surveillance of SMM patients, most of them still experienced target organ damage. However, surveillance led to lower disease burden at the time of active myeloma diagnosis, with reduced rates and severity of bone disease, fewer potentially irreversible MDEs and fewer hospital admissions, reflecting a less aggressive clinical presentation. Furthermore, surveillance may have contributed to improved PFS and OS. Further research is warranted on the optimization of surveillance guidelines and the possible utility of screening, perhaps in high-risk populations. Also, the limited ability of surveillance in the prevention of target organ damage should be recognized in the context of emerging data on early treatment of high-risk SMM.

## Figures and Tables

**Table 1:** Demographic and clinical characteristics of the patients at diagnosis of active MM.

Characteristic	Post SMM (*n* = 57)	DeNovo MM (*n* = 57)	*p* Value
Demographics			
Gender, male—No. (%)	23 (43)	31 (57)	0.09
Age at Active MM diagnosis (yr.), Median (range)	70.5 (42.9–92.4)	70.6 (42.7–88)	0.94
Year of Active MM Diagnosis (Prior 2020/After 2020)	28/29	27/30	
Age at SMM diagnosis (yr.), Median (range)	71 (38–92)	NA	
Year of SMM diagnosis (2006–2014/2015–2018/2019–2022)	17/24/16	NA	
High-Risk SMM—%	67	NA	
Myeloma Diagnosis Criteria *			
CRAB—No. (%)	45 (79)	53 (93)	0.04
Hypercalcemia—No. (%)	1 (2)	7 (12)	0.03
Renal insufficiency—No. (%)	9 (16)	11 (19)	0.4
Anemia—No. (%)	34 (60)	31 (54)	0.8
Bone Lesions—No. (%)	26 (45)	38 (67)	0.02
SLiM Only—No. (%)	9 (16)	2 (3.5)	0.053
≥60% Clonal PC in BM—No. (%)	11 (19)	20 (35)	0.057
Serum FLC ratio ≥ 100—No. (%)	14 (25)	13 (23)	0.67
≥ 1 Focal Lesion † (>5 mm)—No. (%)	7 (12)	12 (21)	0.31
Active Myeloma Characteristics			
Heavy Chain IgG, (%)	17 (30)	40 (70)	0.11
Light Chain Kappa, (%)	28 (49)	29 (51)	0.68
BMPC (%)	35%	48%	0.01
M Protein, g/dL (Median, IQR)	3.6, 0.4–17.5	2.3, 0–7.1	0.28
Involved sFLC, mg/L (Median, IQR)	153, 39–619	294, 58–1086	0.11
High-Risk Genetics ‡ (%)	18 (32)	20 (35)	0.69
Extra Medullary Disease (%)	3 (5)	7 (12)	0.16
ISS I/II/III, (*n*)	22/3/8	15/18/13	0.003
ISS ≥ II (%)	19 (33)	38 (67)	0.003
R-ISS I/II/III, (*n*)	14/7/5	6/25/7	0.002
R-ISS ≥ II (%)	26 (46)	48 (84)	0.001

BMPC Bone Marrow Plasma Cell, FLC Free light chain, ISS International staging system, MM Multiple Myeloma, SMM Smoldering multiple myeloma. Note: Clinical and laboratory values for both cohorts reflect data captured at the time of active myeloma diagnosis, specifically at Cycle 1, Day 1 of therapy. * Active MM was defined as the initiation of systemic therapy; in 5 cases (3 pSMM, 2 dnMM), treatment was started based on an evolving pattern of disease and clinical judgment to prevent imminent organ damage, despite not yet fulfilling all SLiM-CRAB criteria. † Focal bone lesions were assessed using whole-body MRI or PET/CT and were defined as discrete areas of infiltration measuring at least 5 mm in diameter. ‡ High-risk cytogenetics were defined according to IMWG criteria and included the presence of t(4; 14), t(14; 16), t(14; 20), del(17p), or gain(1q).

**Table 2:** Treatment patterns and response.

Characteristic	Post SMM (*n* = 57)	DeNovo MM (*n* = 57)	*p* Value
Regimen			0.58
Doublet	13 (23)	12 (21)	
Triplet	28 (50)	33 (59)	
Quadruplet	12 (21)	11 (20)	
Bortezomib—No. (%)	44 (77)	54 (95)	0.007
Dexamethasone—No. (%)	57 (100)	56 (98)	0.31
Cyclophosphamide—No. (%)	13 (23)	13 (23)	1
Lenalidomide—No. (%)	30 (52.6)	34 (57)	0.57
Thalidomide—No. (%)	3 (5)	4 (7)	0.69
Daratumumab—No. (%)	16 (28)	9 (16)	0.11
ASCT—No. (%)	14 (25)	18 (32)	0.53
Complications at Active MM Presentation			
Required Admission (*n*, %)	5 (9)	18 (33)	0.01
Pain Management—No.	0	11	0.01
Dialysis—No.	1	1	1
Surgical Intervention *—No.	1	7	0.03
Transfusion—No.	1	2	1
Antibiotics †—No.	0	6	0.01
Admission length—Days (Median, range)	6 (3–36)	9 (2–26)	0.63
Response to 1st Line Treatment			
ORR (%)	86	87	1
≥VGPR (%)	56	56	0.88

ASCT Autologous stem cell transplantation, ORR Overall response rate, VGPR very good partial response. * Surgical intervention includes both orthopedic procedures and spinal neurosurgery related to multiple myeloma complications. † Antibiotics refers to treatment for active infection upon presentation.

## Data Availability

The data presented in this study are available on request from the corresponding author. The data are not publicly available due to patient privacy and ethical restrictions.
